# Long-term caloric restriction in *ApoE*-deficient mice results in neuroprotection via Fgf21-induced AMPK/mTOR pathway

**DOI:** 10.18632/aging.101086

**Published:** 2016-11-29

**Authors:** Claire Rühlmann, Tjark Wölk, Tobias Blümel, Laura Stahn, Brigitte Vollmar, Angela Kuhla

**Affiliations:** ^1^ Institute for Experimental Surgery, Rostock University Medical Center, 18057 Rostock, Germany

**Keywords:** *ApoE-*deficiency, caloric restriction, Fgf21, pAMPK, mTOR, Tau-phosphorylation, Alzheimer's disease

## Abstract

Caloric restriction (CR) decelerates the aging process, extends lifespan and exerts neuroprotective effects in diverse species by so far unknown mechanisms. Based on known neuroprotective effects of fibroblastic growth factor 21 (Fgf21) we speculate that CR upregulates Fgf21, which phosphorylates neuronal AMP-activated protein kinase (AMPK), leading to a decrease of mammalian target of rapamycin (mTOR) signaling activity and an inhibition of tau-hyperphosphorylation. This in turn reduces the formation of neurofibrillary tangles, a neuropathological hallmark of Alzheimer's disease. *ApoE*-deficient mice (*ApoE−/−*), serving as a model of neurodegeneration, showed upon CR vs. ad libitum feeding increased Fgf21 levels in both, plasma and brain as well as higher phosphorylation of fibroblastic growth factor receptor 1c (Fgfr1c), extracellular signal-regulated kinases 1/2 (ERK1/2) and AMPK in brain, lower activity of mTOR and decreased Tau-phosphorylation. Finally, CR in *ApoE−/−* mice caused neuroprotection as indicated by a higher synaptic plasticity shown by immunohistochemical analysis with increased numbers of PSD95-positive neurons and a better cognitive performance as analyzed with Morris water maze test. These data provide substantial evidence that neuroprotection upon CR seems to be Fgf21-dependent. Further experiments are necessary to evaluate Fgf21 as a therapeutic tool to treat tauopathy for improvement of cognitive performance.

## INTRODUCTION

In all species studied to date, restricted calorie intake by 20-50% while providing adequate micronutrient supply significantly extends mean and maximal lifespan [1, 2]. Moreover, age-related deficits in learning and motor coordination are ameliorated by caloric restriction (CR) in rodents [3, 4]. In line with this, CR attenuates amyloid deposition in monkeys and in transgenic mouse models of Alzheimer's disease (AD) [5, 6], leading to improvement of cognitive deficits [7] and to reduction of neuronal loss in neocortex, hippocampus, and striatum [8]. Further, it is described that CR promotes neurogenesis in adult rodents, probably by increasing brain-derived neurotrophic factor levels [9]. However, the underlying mechanisms in response to CR remain unclear.

Recently, the fibroblastic growth factor 21 (Fgf21) was described as starvation hormone [10]. Fgf21 is upregulated in response to CR in the liver and is secreted into plasma [11]. Fgf21 activity occurs when Fgf21 binds to fibroblast growth factors receptor (Fgfr) and β-klotho, a single transmembrane protein [12]. Fgfrs consist of seven major isoforms (1b, 1c, 2b, 2c, 3b, 3c and 4), whereby the isoform Fgfr1c is the primary receptor of Fgf21 in the mediation of its activity in *in vivo* studies [13]. When Fgf21 binds to its receptor, it leads to a rapid phosphorylation of downstream pathway components, including the MAPK cascade [14] and results via protein kinase A to activation of AMP-activated protein kinase (AMPK) [15]. In addition, *fgf21* is also a direct target gene of the peroxisome proliferator-activated receptor-α (*pparα*) [16, 17], a regulator for CR-induced lipolysis. Therefore, Fgf21 plays an important role in adaptation to metabolic states, which require increased fatty acid oxidation and ketogenesis [18] as an alternative energy source [17]. Ketogenesis, which is basically triggered by Fgf21, leads to AMPK activation not only in the periphery [19] but also in the central nervous system [20], resulting in decreased mammalian target of rapamycin (mTOR) signaling [21, 22]. It could additionally be shown that inhibition of the mTOR pathway with rapamycin protects the entorhinal cortex from Tau-mediated neurodegeneration [23].

Since plasma Fgf21 can cross the blood brain barrier by simple diffusion and can be detected in human cerebrospinal fluid and brain tissues in rodents [24, 25], it is conceivable to assume that Fgf21 contributes to regulation of brain metabolism. There is growing evidence that Fgf21 has neuroprotective effects and improves cognition [26]. It could be shown that incubation of human dopaminergic neurons with Fgf21 resulted in enhanced mitochondrial function and mitochondrial respiratory capacity [27]. Based on the neuronal functional activity of Fgf21 we speculate that Fgf21 phosphorylates neuronal AMPK. Activated AMPK leads to decrease of mTOR signaling activity [21] and in consequence to inhibition of Tau-hyperphosphorylation with reduction of neurofibrillary tangles (NFTs) [28] as a neuropathological hallmark of AD.

Apolipoprotein E (ApoE) binds to Tau-protein and prevents its hyperphosphorylation [29], leading to slow down of NFTs-formation. *ApoE*-deficient mice (*ApoE−/−*) represent a well-established mouse model of tauopathy [30] with memory deficits [31]. In addition, *ApoE−/−* compared to wild-type mice revealed throughout life a 2- to 7-fold lower expression of hepatic *fgf21* (own unpublished data). Since Fgf21 has neuroprotective properties, it may be assumed that low Fgf21 contributes to neurodegeneration. To pursue this issue, we fed *ApoE−/−* mice caloric-restricted for a long-term to raise hepatic as well as neuronal Fgf21 with the aim to prevent tauopathy via the AMPK/mTOR pathway and to improve cognitive performance.

## RESULTS

### Long-term CR slowed increase of body weight in *ApoE−/−* mice

**Figure 1:**
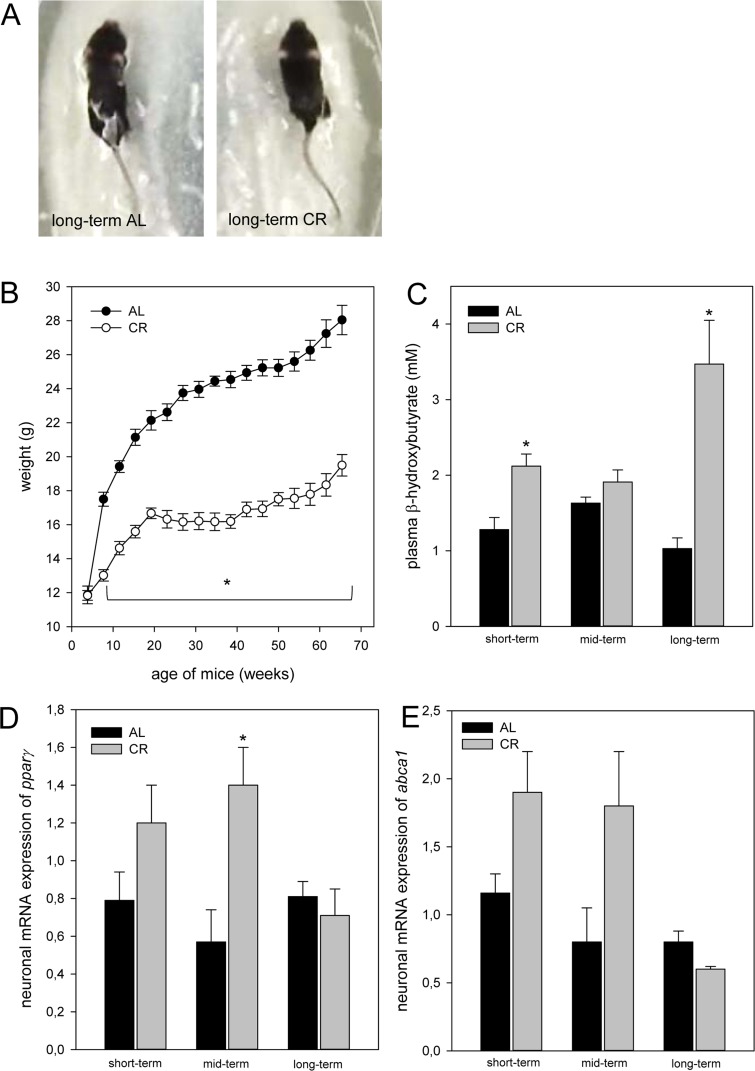
(**A**) Image of one long-term ad libitum (AL)- and of one caloric-restricted (CR)-fed *ApoE−/−* mouse. These mice were fed either AL or CR (60% of ad libitum). These images exemplarily show that in general CR-fed mice were smaller in body size than AL-fed mice. (**B**) Body weight (g) of AL- and CR-fed mice over a period of 68 weeks. In general, parameter of ketogenesis and lipolysis are increased in CR-fed mice when compared with the age-matched AL-fed mice indicated by a marked rise of (**C**) plasma β-hydroxybutyrate and of neuronal (**D**) *pparγ* and (**E**) *abca1* mRNA expressions. Values are given as mean±SEM; ANOVA, post-hoc pairwise comparison tests: * p < 0.05 vs. AL.

In general, CR-fed mice were smaller in body size than the ad libitum (AL)-fed mice (Fig. 1A). The body weight of AL-fed *ApoE−/−* mice continuously increased 2-fold with aging up to 28.10 ± 0.85 g. Long-term CR resulted in a very slow increase of body weight reaching values of 19.6 ± 0.63 g. In general, at all time points CR-fed mice showed significantly lower levels of body weight when compared to AL-fed mice (Fig. 1B).

### CR increased ketogenesis and neuronal lipolytic gene expression in *ApoE−/−* mice

CR-fed mice revealed a continuous rise of ketone bodies, as given by an up to 2-fold increase of plasma β-hydroxybutyrate concentrations in long-term-fed mice when compared to short-term-fed mice. On the contrary, the concentrations of β-hydroxybutyrate remained almost unchanged in AL-fed mice averaging at low values of 1 mM up to 1.6 mM (Fig. 1C). Ketogenesis was significantly higher in CR- than in AL-fed *ApoE−/−* mice after short- and long-term feeding. The neuronal mRNA expression of *pparγ* and *abca1* remained unchanged with aging in AL-fed *ApoE−/−* mice (Fig.1. D and E) while short- and mid-term CR markedly increased the neuronal mRNA expression of *pparγ* and *abca1* (Fig. 1 D and E).

### CR increased hepatic expression and systemic concentration of Fgf21 in *ApoE−/−* mice

**Figure 2:**
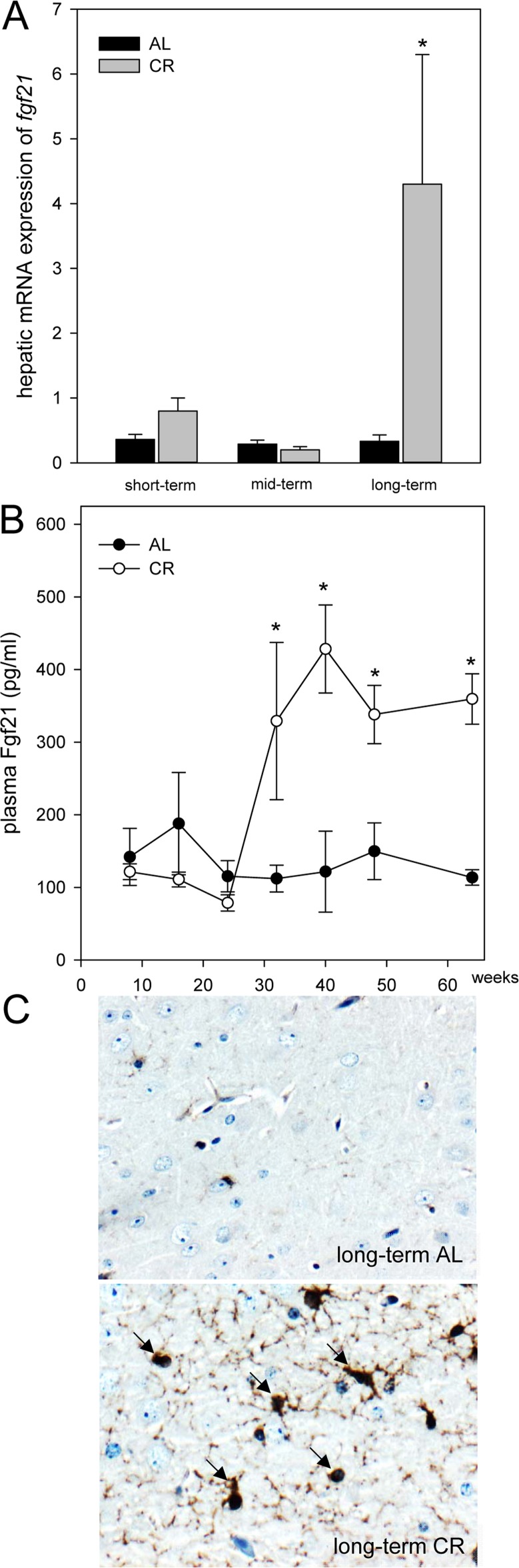
(**A**) Quantitative real-time PCR analysis of hepatic mRNA expression of *fgf21* and (**B**) quantitative analysis of plasma Fgf21 of *ApoE−/−* mice. Mice were fed either ad libitum (AL) or caloric-restricted (CR, 60% of ad libitum) for a short-term (4 weeks; n=14), mid-term (20 weeks; n=14) or long-term (64 weeks; n=14). At weeks 8, 16, 24, 32, 40, 48 and 64 plasma Fgf21 was measured. Signals were corrected to that of RPS18. Representative immunohistochemical images (**C**, original magnification x400) of Fgf21 accumulation in brain of long-term AL- (upper panel) and CR-fed *ApoE−/−* mice (lower panel) mice. Values are given as means ± SEM; ANOVA, post-hoc pairwise comparison tests.* p < 0.05 vs. AL.

**Figure 3:**
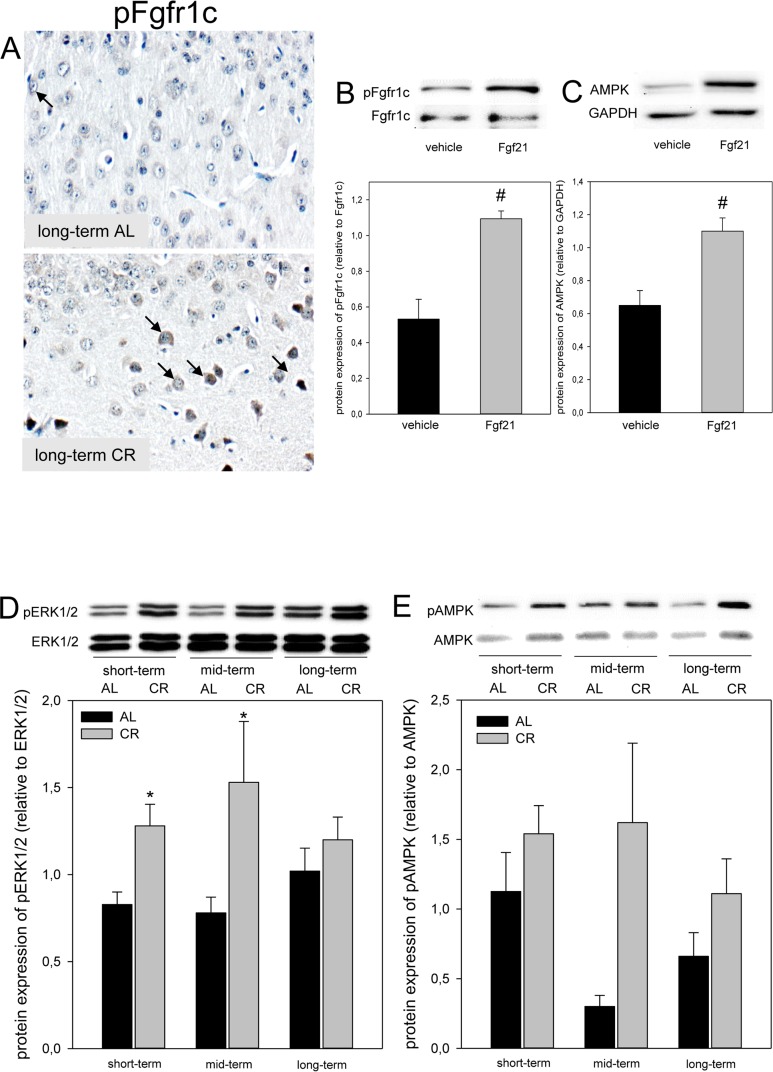
(**A**) Representative immunohistochemical images (original magnification x400) of pFgfr1c expression in brain of long-term ad libitum- (AL, upper panel, indicated by arrows) and of caloric-restricted-fed (CR, lower panel, indicated by arrows) *ApoE−/−* mice. Representative Western blots as well as densitometric analysis of (**B**) pFgfr1c and (**C**) AMPK expression in primary glial cells, which were treated with vehicle (DMEM/F12) and 5 μg/ml Fgf21. Representative Western blot and densitometric analysis of (**D**) pERK1/2 and (**E**) pAMPK expression in brain of *ApoE−/−* mice. Mice were fed either AL or CR (60% of ad libitum) for a short-term (4 weeks; n=14), mid-term (20 weeks; n=14) or long-term (64 weeks; n=14). Signals were corrected to that of either ERK1/2 or AMPK. Values are given as means ± SEM; ANOVA, post-hoc pairwise comparison tests: * p < 0.05 vs. AL, # p < 0.05 vs. vehicle.

Of note, the hepatic mRNA expression of *fgf21* in *ApoE−/−* mice was significantly increased upon long-term CR (Fig. 2A). Accordingly, the systemic Fgf21 concentration in *ApoE−/− mice* raised significantly and reached approx. 3-fold higher levels upon long-term CR when compared to AL feeding (Fig. 2B). Fgf21 was barely measureable in the brain of *ApoE−/−* mice (Fig. 2C; upper panel) but was detectable at a much higher level upon a long-term CR (Fig. 2C; arrows, lower panel) with a preferential location around glial cells in the cortex. Along with the higher neuronal Fgf21 levels upon long-term CR, the receptor for Fgf21, namely Fgfr1c, was activated, as indicated by an increased number of pFgfr1c-positive neuronal cells in the cortex (Fig. 3A; lower panel, arrows).

### CR increased Fgfr1c downstream signaling in *ApoE−/−* mice

Next we asked whether Fgf21 activates the neuronal downstream signaling pathway via Fgfr1c *in vitro* and could show that phosphorylation of Fgfr1c is significantly increased in Fgf21-treated primary glial cells compared to vehicle alone (Fig. 3B). Along with activation of Fgfr1c the AMPK protein expression was significantly increased in Fgf21-treated primary glial cells compared to vehicle alone (Fig. 3C). Further, a significant increase of ERK1/2-phosphorylation was found upon short- as well as mid-term CR vs. AL feeding in *ApoE−/−* mice (Fig. 3D). In addition, the phosphorylation of AMPK was consistently increased upon CR (Fig. 3E).

### CR inhibited mTOR and Tau-phosphorylation in *ApoE−/−* mice

**Figure 4:**
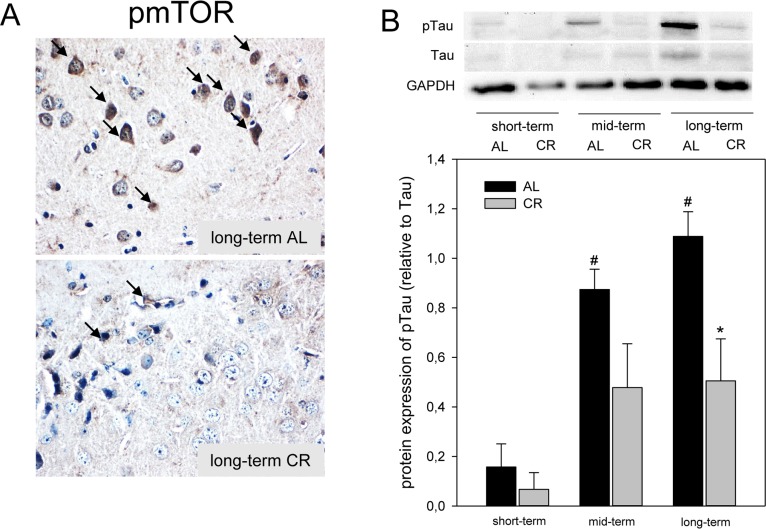
(**A**) Representative immunohistochemical images (original magnification x400) of pmTOR expression in brain of long-term ad libitum- (AL, upper panel) and of caloric-restricted-fed (CR, lower panel) *ApoE−/−* mice. (**B**) Representative Western blot and densitometric analysis of pTau in brain of *ApoE−/−* mice. Mice were fed either AL or CR (60% of ad libitum) for a short-term (4 weeks; n=14), mid-term (20 weeks; n=14) or long-term (64 weeks; n=14). Signals were corrected to Tau. GAPDH served as loading control. Values are given as means ± SEM; ANOVA, post-hoc pairwise comparison tests: * p < 0.05 vs. AL.

Since activation of AMPK inhibits mTOR, we analyzed the phosphorylation of mTOR by immunohisto-chemistry and could show that long-term CR reduced the activity of mTOR in the cortex of *ApoE−/−* mice, as indicated by a decreased number of pmTOR-positive neuronal cells (Fig. 4A; lower panel, arrows). It could further be demonstrated that an inhibition of mTOR upon CR feeding resulted in an almost 2-fold reduction of Tau-phosphorylation at all time points, which was significant upon long-term CR (Fig. 4B) while AL-fed mice revealed an age-dependent increase of Tau-phosphorylation (Fig. 4B).

### Long-term CR increased working memory and neuronal plasticity in *ApoE−/−* mice

**Figure 5:**
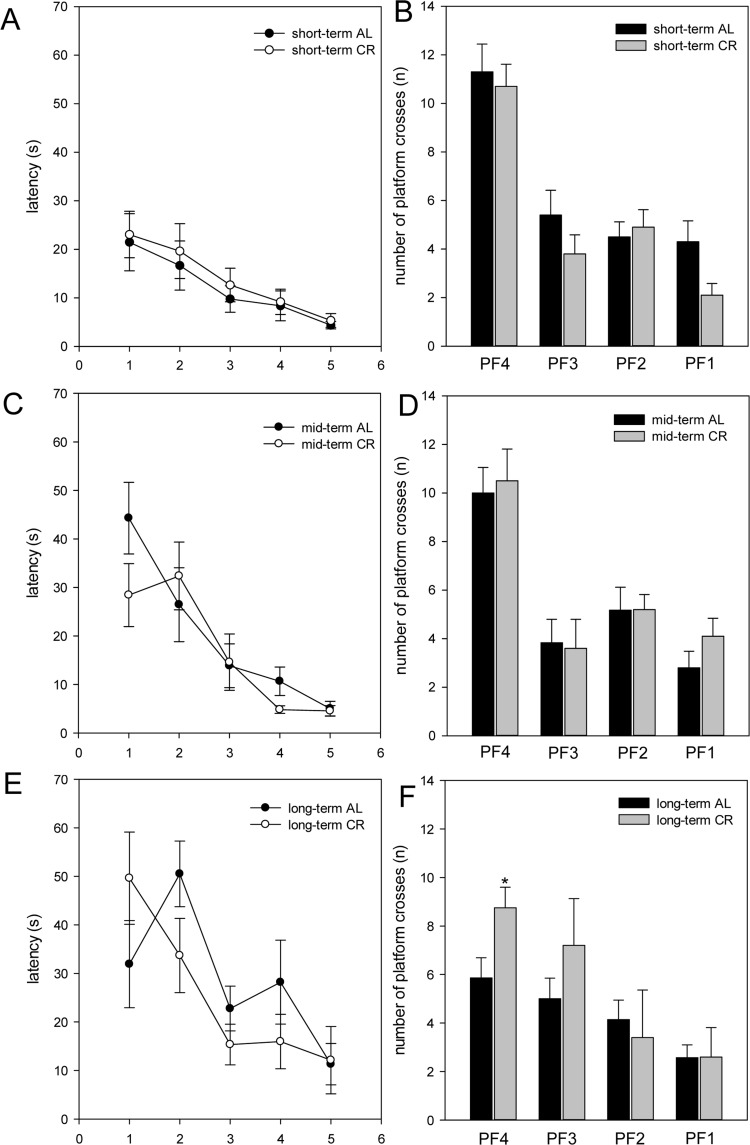
The escape latencies (in s) of the training were displayed in five blocks (**A, C, E**; x-axis 1-5). One block consisted of four consecutive trials. The escape latencies decreased throughout the training days. In the test trial, the number of platform crossings during 60 s was measured (**B, D, F**). *ApoE−/−* mice were fed either ad libitum (AL) or caloric-restricted (CR, 60% of ad libitum) for a short-term (**A** and **B**, 4 weeks; n=14), mid-term (**C** and **D**, 20 weeks; n=14) or long-term (**E** and **F**, 64 weeks; n=14) and were trained and tested 5 days before being sacrificed. Values are given as mean±SEM; ANOVA, post-hoc pairwise comparison tests: *p < 0.05 vs. AL.

Mice were tested in the spatial reference memory version of the Morris water maze. AL- and CR-fed *ApoE−/−* mice did not show significant differences in general swimming performance, which was determined by measurement of the swimming speed during the test trials (data not shown). Escape latencies were displayed in five blocks. Each block consisted of four consecutive trials. It is expected, escape latencies decreased throughout the training days. Thus, all mice were able to learn the task, whereas aged mice (long-term AL/CR) vs. younger mice (short- and mid-term AL/CR) started with a *per se* inferior performance (Fig. 5A, C, E). While short- and mid-term-fed mice of both groups revealed almost comparable escape latency, long-term CR resulted in lower escape latency found for block 2 - 4 (Fig. 5E), suggesting that long-term CR caused a better training performance due to better cognitive activities.

All mice were exercised to the platform 4 (PF4) during training. Finally, the platform was removed and the number of platform crossings during 60 s was measured. Thereby, it could be shown that short- as well as mid-term-fed mice of both groups crossed PF4 nearly with the same frequency (Fig. 5B, D, F). The most striking differences between the AL- and CR-fed mice were found in the number of platform crossings of long-term-fed mice. Long-term CR-fed mice crossed PF3 up to 30% and PF4 significantly more often than AL-fed mice (Fig. 5F). Thus, long-term CR resulted in a better working memory.

### CR delayed the decrease of neuronal PSD95 expression in *ApoE−/−* mice

**Figure 6:**
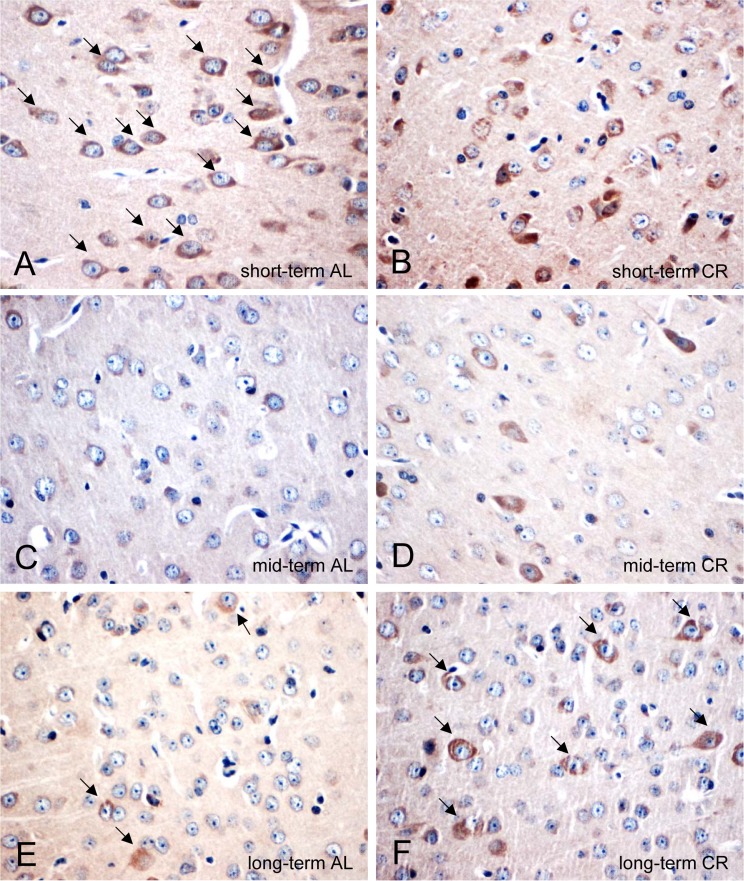
(**A-F**) Representative immunohistochemical images (original magnification x400) of PSD95 protein expression in brain of short-term (**A** and **B**), mid-term (**C** and **D**) and long-term (**E** and **F**) ad libitum- (AL, left panels) and caloric-restricted-fed (CR, right panels) *ApoE−/−* mice. Mice were fed either AL or CR (60 % of ad libitum) for a short-term (4 weeks; n=14), mid-term (20 weeks; n=14) or long-term (64 weeks; n=14). Note the decrease of PSD95-positive neurons in the AL-fed *ApoE−/−* mice while a long-term CR delayed the decline of PSD95-positive neurons in the ApoE−/− mice (indicated by arrows).

AL-fed *ApoE−/− mice* revealed an age-dependent decrease of the number of PSD95-positive neuronal cells (Fig. 6A, C, E; arrows) while this could not be observed in CR mice, as shown by an almost stable expression of PSD95 (Fig. 6B, D, F; arrows).

## DISCUSSION

The main finding of the present study is that long-term CR in *ApoE−/−* mice increased lipolysis and ketogenesis in the animals. This was reflected by higher systemic ketone body concentrations and increased neuronal mRNA expressions of *pparγ* and *abca1*. CR further caused both, a systemic and neuronal increase of Fgf21, leading to higher activation of Fgfr1c in the brain and, in consequence, to activation of the AMPK/mTOR pathway. AMPK-induced inhibition of mTOR in CR-fed *ApoE−/−* mice reduced Tau-phosphorylation and improved their cognition performance.

Most recently, we could demonstrate that long-term CR i.e. starting at the age of weaning until pre-senescence reduced the normal age-related decline in cognitive performance [4]. This underlines the importance of lifestyle factors such as nutrition in the context of metabolic syndrome-associated neurodegeneration [32]. However, the mechanism of CR-induced neuroprotection is not yet completely understood. To address this issue, we used *ApoE−/−* mice an accepted model of metabolics-driven neurodegeneration. The animals were maintained under CR for a long-term to evaluate neuroprotective molecular mechanisms. *ApoE−/−* mice are known for their significant loss of synapses with increasing age [33] and further are also described as model of tauopathy [30].

In response to fasting, lipolysis and β-oxidation are enhanced to provide alternative energy resources for the organism [17, 34]. In addition, marked elevations in Fgf21 expression were observed in mice upon fasting [18] and food restriction [11]. Elevated *fgf21* gene expression and serum Fgf21 concentrations in food-restricted animals were accompanied by an upregulation of *pparα* mRNA expression [11]. PPAR*α* regulates the hepatic lipid metabolism by induction of ketogenesis and lipolysis via increased β-oxidation [11, 35]. Accordingly, the present study shows an accumulation of ketone bodies upon CR. Ketone bodies may act neuroprotective [36] and thereby, enhance memory performance [37]. In addition, we found that lipolysis and β-oxidation are also enhanced in brain as shown by increased neuronal mRNA expression of *pparγ* and of its target gene *abca1*. Since activation of PPARγ reduces AD-pathology and improves cognitive function in mouse models of AD [38] this current finding might also contribute to neuroprotection.

Fgf21 is present in the brain [39] including midbrain and *in vitro* is expressed by glial cells [27]. Mäkelä and coworkers found additionally that Fgf21 plays a regulatory role in brain energy metabolism as shown by enhanced mitochondrial capacity in Fgf21-treated dopaminergic neurons [27]. Further, it is known that Fgf21 can cross the blood brain barrier [24]. Thus, it is conceivable to state that CR-induced hepatic Fgf21 might be responsible for the positive immunostaining of glial cells *in vivo*, as observed in the present study (Fig. 2C). Fgfr1c, the receptor for Fgf21, is found to be expressed in hypothalamic region of the brain [40] and in cortical neurons (current study; Fig. 3A). Activation of Fgfr1c -most occasionally via Fgf21-positive glial cells- phosphorylates specific tyrosine residues that mediate interaction with cytosolic adaptor proteins and intracellular signaling pathways [14]. This view is supported by our *in vitro* data showing the activation of Fgfr1c and the increase of downstream AMPK protein upon Fgf21 exposure of glial cells and by our *in vivo* data showing increased Fgfr1c- as well as increased ERK1/2- and AMPK-phosphorylation in CR-fed mice. Since Fgf21 is capable to elevate phosphorylation of AMPK [19], we assume that CR-induced rise of Fgf21 triggers the AMPK pathway. However, it has to be mentioned that conditions of perceived energy deprivation, such as fasting, food restriction, and exercise, increase the AMP/ATP ratio, eventually causing an activation of AMPK [41 and current study].

AMPK is a suppressor of mTOR signaling [42]. Abrogation of TOR signaling causes rejuvenation in the *C. elegans* [43]. This new function for TOR signaling in ageing control may represent a link between nutrition, metabolism and longevity [43]. In support of this function, inhibition of mTOR is described to be protective in neurodegenerative diseases such as AD by enhancement of autophagy, a biological process that not only facilitates the clearance of mutant proteins but also significantly reduces the build-up and accumulation of toxic protein aggregates such as NTFs, being caused by Tau-hyperphosphorylation [44, 45]. Therefore, reduced mTOR and Tau-phosphorylation in the long-term CR-fed *ApoE −/−* mice can be most supposedly attributed to the activation of AMPK pathway by increased Fgf21. In parallel, Ma and coworkers showed that food restriction could lead to activation of SIRT1 and suppression of mTOR activation in mice and speculated that the SIRT1/mTOR signaling pathway may be involved in the neuroprotective effect of food restriction [46]. This view is supported by the study of Guo et al. [47], which demonstrated that food restriction-induced SIRT/mTOR activation contributed to brain integrity. In addition, positive properties of SIRT signaling in *ApoE−/−* mice was reported by the groups of Stein et al. [48] and Palacios et al. [49]. These studies showed that of SIRT-overexpression diminishes atherogenesis and that diet- and exercise-induced SIRT/AMPK pathway regulates muscle energy homeostasis.

Moreover limited Tau-phosphorylation by Fgf21-dependent AMPK/mTOR signaling, improved cognitive performance upon long-term CR restriction might also be due to maintenance of PSD95-positive cells and thus, neuronal plasticity.

In summary, these data provide substantial evidence that neuroprotection upon CR seems to be Fgf21/AMPK/mTOR-dependent. Further experiments are necessary to evaluate Fgf21 as a therapeutic tool to treat tauopathy for improvement of cognitive performance.

## MATERIALS AND METHODS

### Animals

Female Apolipoprotein (*Apo) E* deficient (*ApoE−/−*) mice at the age of 4 weeks were fed either ad libitum (AL) or caloric-restricted (CR, 60% of ad libitum) for 4 additional weeks (short-term, n=10 for each group), 20 weeks (mid-term, n=10 for each group) or 64 weeks (long-term, n=10 for each group). All mice were housed in standard cages in a temperature-controlled room (22°C±2°C) on a 12 h light/dark cycle with free access to food (4.2% fat) and water under specific pathogen free (SPF) conditions. The mice were weighed weekly and directly before they were sacrificed. The experimental protocol was approved by the local Animal Research Committee (Landesamt für Landwirtschaft, Lebensmittelsicherheit und Fischerei (LALLF) of the state Mecklenburg-Western Pomerania (LALLF M-V/TSD/7221.3-1.1-002/14)) and all animals received care according to the German legislation on protection of animals and the Guide for the Care and Use of Laboratory Animals (NIH publication 86-23 revised 1985).

### Sampling and assays

For the analysis of Fgf21-kinetics blood samples were taken from all mice at weeks 8, 16, 24, 32, 40, 48 and 64. At the end of the experiment, all mice were exsanguinated by puncture of the vena cava inferior for immediate separation of plasma, followed by harvest of brain and liver tissues. Measurements of Fgf21 and ketone bodies in plasma were performed using the Fgf21 immunoassay and β-hydroxybutyrate assay kit methods according to the manufacturer's instructions (Fgf21: R&D System; β-hydroxybutyrate: Cayman Chemical Company).

### Glial cell culture

Primary glial cells were cultured from postnatal day 0-3 C57BL/6J mice as described previously [50]. First, pups were decapitated, brains isolated in cold PBS (Gibco) with 1% penicillin/streptomycin (Invitrogen), and 0.1% glucose (Sigma), cerebellum, the meninges, and blood vessels were removed from the cortices. The latter were then minced and trypsinized with 0,05% trypsin-EDTA (Merck Millipore), 0.1% glucose (Sigma), 1% penicillin/streptomycin (Invitrogen) and DNAse (10 μg/mL) for 10 min at 37°C. To stop trypsin digestion, DMEM/F12 (Merck Millipore) containing 10% heat-inactivated fetal bovine serum (HI-FBS, PAA) with supplement 1% penicillin/streptomycin was added. Cells were triturated, filtrated and centrifuged for 5 min at 200 xg. Supernatant was discarded and the pellet resuspended in DMEM/F12 containing 15% (HI-FBS) and 1% penicillin/streptomycin. Cortical cells were seeded in DMEM/F12 with 15% HI-FBS and 1% penicillin/streptomycin at a density of 2*10^6^/well in a 6 well-plate (Cellstar, Greiner bio-one). Medium was changed the following day to fresh DMEM/F12 with 10% HI-FBS and 1% penicillin/streptomycin and incubated for 14-21 d at 37°C and 5% CO_2_. Medium was replaced every 4-5 d. Afterwards cells were exposed with 5 μg/ml Fgf21 in medium or medium as vehicle and incubated for 24 h at 37°C, and 5% CO_2_. Subsequently, cells were harvested for protein analysis.

### RNA analysis of brain and liver tissue

Total RNA was isolated using the RNeasy^®^ Mini Kit (Qiagen) in accordance with the manufacturer's instructions. 2 μg of total RNA was reverse-transcribed with SuperScript ^TM^ First Strand Synthese System (Invitrogen) as described by the manufacturer. Real-time quantitative PCR assays were performed by using Lightcycler 1.5 using the Lightcycler^®^ FastStart DNA Master^Plus^ SYBR Green I kit (Roche Diagnostics). Each amplification mixture (20 μl) contained 5 μM primer, 19 μl of universal PCR Mastermix, and 1μl 1:2 diluted cDNA solution. PCR thermocycling parameters were 95°C for 10 min and 40 cycles of 95°C for 10 s, 55°C for 5 s and 72°C for 10 s. All samples were analyzed for ribosomal protein S18 (RPS18) expression. For analysis of the relative change in gene expression we used the 2^−ΔΔCt^ method. A cDNA pool of livers of untreated C57BL/6J mice served as the control and therefore as the first Δ. The second Δ is represented by Ct-values of RPS18 amplification. Specificity of the amplification was verified by melt-curve analysis and evaluation of efficiency of PCR amplification. The primers are listed in Table 1.

**Table 1:** 

Transcript	forward primer	reverse primer
***pparγ***	5′-TCATGACCAGGGAGTTCCTC-3′	5′-CAGGTTGTCTTGGATGTCCTC-3′
***abca1***	5′-ACTGGAGACACCCCTGTGAC-3′	5′-GGAGAGCTTTCGTTTGTTGC-3′
***fgf21***	5′-GCTGTCTTCCTGCTGGGG-3′	5′-CCTGGTTTGGGGAGTCCTTC-3′
***rps18***	5′-AGGATGTGAAGGATGGGAAG-3′	5′-TTGGATACACCCACAGTTCG-3′

### Western Blot analysis of brain tissue and of glial cells

Harvested brain tissues and glial cells were further processed for protein isolation. For this purpose, brain tissue and glial cells were homogenized in lysis buffer (10 mM Tris pH 7.5, 10 mM NaCl, 0.1 mM EDTA, 0.5% Triton-X 100, 0.02% NaN_3_, and 0.2 mM PMSF (a protease inhibitor cocktail), incubated for 30 min on ice and centrifuged for 10 min at 4°C and 10.000 xg. Protein contents were assayed by the bicinchoninic acid method (Pierce Biotechnology) with 2.5% BSA (Pierce Biotechnology) as standard. On 12% SDS gels, 20 μg (pTau, Tau, pERK1/2 and ERK1/2) and 40 μg (pAMPK and AMPK) protein from brain tissue was separated and transferred to a polyvinyldifluoride membrane (Immobilon-P; Millipore). On 8% SDS gels, 20 μg (pFgfr1c, Fgfr1c and AMPK) protein from glial cells was separated and transferred to a polyvinyldifluoride membrane (Immobilon-P; Millipore). After blockade with 2.5% BSA (Pierce Biotechnology), membranes were incubated overnight at 4°C with a mouse monoclonal anti-pAMPK (1:1.000), a rabbit polyclonal anti-AMPK (1:1.000), a mouse monoclonal anti-pTau (1:1.000), a mouse monoclonal anti-Tau (1:1.000), a rabbit polyclonal anti-pFgfr1c (1:1.000), a rabbit monoclonal anti-Fgfr1c (1:1.000), a rabbit monoclonal anti-ERK1/2 (1:1.000), or a rabbit polyclonal anti-pERK1/2 (1:1.000), respectively. Afterwards, a secondary peroxidase-linked horse anti-mouse (pAMPK, AMPK, pTau, Tau; 1:10.000) or a goat anti-rabbit (pFgfr1c, Fgfr1c; 1:10.000; pERK1/2, ERK1/2; 1:5.000) was applied. All antibodies were supplied by Cell Signaling. Protein expression was visualized by means of luminol-enhanced chemiluminescence (ECL plus; Amersham Pharmacia Biotech) and digitalized with ChemiDoc™ XRS System (Bio-Rad Laboratories). Signals were densitometrically assessed (Quantity One; Bio-Rad Laboratories) and densities normalized either to GAPDH (mouse monoclonal anti-GAPDH antibody; 1:20.000; Millipore) or to unphosphorylated proteins (AMPK, ERK1/2, Tau, Fgfr1c).

### Immunohistology

For immunohistochemical analysis, brain tissue was fixed in 4% phosphate-buffered formalin for 5-6 weeks and subsequently embedded in paraffin. From the paraffin-embedded tissue blocks, 4 μm thin sections were put on X-tra Adhesive Precleaned Micro Slides (Leica) and exposed to a rabbit monoclonal anti-Fgf21 antibody (1:500, Abcam), rabbit polyclonal anti-pFgfr1c antibody (1:100, Cell Signaling), rabbit monoclonal anti-pmTOR (IHC specific, 1:100, Cell Signaling) and rabbit polyclonal anti-PSD95 (1:1000, Abcam). For the development of the primary antibodies with DAB chromogen Universal LSAB^®^ kits (System-HRP; DakoCytomation, Dako) were used according to the manufacturer's instructions. The sections were counterstained with hemalaun and analyzed with a light microscope (Olympus BX51). Images were acquired with a Color View II FW camera (Color View).

### Morris water maze test

The Morris water maze (MWM) was performed as a task to measure spatial reference memory. Mice under dim light conditions (indirect illumination, 3.0 Lux) at days −4 to −1 d were trained to locate a submerged hidden platform (11 cm diameter) in a fixed quadrant of a circular pool filled with opaque water (diameter of pool 107 cm, filled to a depth of 20 cm, water temperature 17º C, platform submerged 1 cm beneath the surface). For each trial (1 x at −4 d to familiarize the animals with test apparatus, 8 x at −3 d, 8 x at −2 d, 4 x −1 d = 5 training blocks each consisting of 4 consecutive trials) mice are gently placed in the water, hind legs first. On each trial, the starting position was randomized between four possible quadrant positions. The location of the platform remained constant throughout the whole training period. Each trial lasted until the animal located the platform. However, after max. 60 s the mouse was guided to the platform. The latter was given a latency score of 60 s. Mice were allowed to rest 10 s on the escape platform, were then removed, dried with a towel and warmed under a heating lamp. After further 30 s the next trial started. The time to reach the platform (latency to escape) was recorded for each trial. On day −1, mice finally were trained for four trials. All training trials were monitored online by a video camera [4]. After training, the platform was removed from the pool and the mouse performed a 60 s test trial. The start position of the test trial was located opposite to the quadrant that had contained the platform. The swimming route and the number of times the mouse crossed each of the four possible platform positions (the previous platform position and 3 imaginary platform position in the other quadrants and platform crossings) was monitored online by a video camera [4].

### Statistical analysis

All data are expressed as means ± SEM. Statistical differences were determined using one- or two-way ANOVA followed by post-hoc pairwise comparison tests for analysis between either feeding groups or duration of feeding. Data were considered significant if p<0.05. Statistical analysis was performed using the SigmaStat software package (Jandel Scientific).

## References

[1] Weindruch R, Walford RL, Fligiel S, Guthrie D (1986). The retardation of aging in mice by dietary restriction: longevity, cancer, immunity and lifetime energy intake. J Nutr.

[2] Mulligan JD, Stewart AM, Saupe KW (2008). Downregulation of plasma insulin levels and hepatic PPARgamma expression during the first week of caloric restriction in mice. Exp Gerontol.

[3] Duan W, Guo Z, Jiang H, Ware M, Mattson MP (2003). Reversal of behavioral and metabolic abnormalities, and insulin resistance syndrome, by dietary restriction in mice deficient in brain-derived neurotrophic factor. Endocrinology.

[4] Kuhla A, Lange S, Holzmann C, Maass F, Petersen J, Vollmar B, Wree A (2013). Lifelong caloric restriction increases working memory in mice. PLoS One.

[5] Patel NV, Gordon MN, Connor KE, Good RA, Engelman RW, Mason J, Morgan DG, Morgan TE, Finch CE (2005). Caloric restriction attenuates Abeta-deposition in Alzheimer transgenic models. Neurobiol Aging.

[6] Qin W, Chachich M, Lane M, Roth G, Bryant M, de Cabo R, Ottinger MA, Mattison J, Ingram D, Gandy S, Pasinetti GM (2006). Calorie restriction attenuates Alzheimer's disease type brain amyloidosis in Squirrel monkeys (Saimiri sciureus). J Alzheimers Dis.

[7] Halagappa VK, Guo Z, Pearson M, Matsuoka Y, Cutler RG, Laferla FM, Mattson MP (2007). Intermittent fasting and caloric restriction ameliorate age-related behavioral deficits in the triple-transgenic mouse model of Alzheimer's disease. Neurobiol Dis.

[8] Roberge MC, Hotte-Bernard J, Messier C, Plamondon H (2008). Food restriction attenuates ischemia-induced spatial learning and memory deficits despite extensive CA1 ischemic injury. Behav Brain Res.

[9] Vasconcelos AR, Yshii LM, Viel TA, Buck HS, Mattson MP, Scavone C, Kawamoto EM (2014). Intermittent fasting attenuates lipopolysaccharide-induced neuroinflammation and memory impairment. J Neuroinflammation.

[10] Zhang Y, Xie Y, Berglund ED, Coate KC, He TT, Katafuchi T, Xiao G, Potthoff MJ, Wei W, Wan Y, Yu RT, Evans RM, Kliewer SA, Mangelsdorf DJ (2012). The starvation hormone, fibroblast growth factor-21, extends lifespan in mice. eLife.

[11] Kuhla A, Hahn S, Butschkau A, Lange S, Wree A, Vollmar B (2014). Lifelong caloric restriction reprograms hepatic fat metabolism in mice. J Gerontol A Biol Sci Med Sci.

[12] Kharitonenkov A (2009). FGFs and metabolism. Curr Opin Pharmacol.

[13] Yang C, Jin C, Li X, Wang F, McKeehan WL, Luo Y (2012). Differential specificity of endocrine FGF19 and FGF21 to FGFR1 and FGFR4 in complex with KLB. PLoS One.

[14] Ornitz DM, Marie PJ (2015). Fibroblast growth factor signaling in skeletal development and disease. Genes Dev.

[15] Cyphert HA, Alonge KM, Ippagunta SM, Hillgartner FB (2014). Glucagon stimulates hepatic FGF21 secretion through a PKA- and EPAC-dependent posttranscriptional mechanism. PLoS One.

[16] Lundåsen T, Hunt MC, Nilsson LM, Sanyal S, Angelin B, Alexson SE, Rudling M (2007). PPARalpha is a key regulator of hepatic FGF21. Biochem Biophys Res Commun.

[17] Badman MK, Pissios P, Kennedy AR, Koukos G, Flier JS, Maratos-Flier E (2007). Hepatic fibroblast growth factor 21 is regulated by PPARalpha and is a key mediator of hepatic lipid metabolism in ketotic states. Cell Metab.

[18] Domouzoglou EM, Maratos-Flier E (2011). Fibroblast growth factor 21 is a metabolic regulator that plays a role in the adaptation to ketosis. Am J Clin Nutr.

[19] Chau MD, Gao J, Yang Q, Wu Z, Gromada J (2010). Fibroblast growth factor 21 regulates energy metabolism by activating the AMPK-SIRT1-PGC-1alpha pathway. Proc Natl Acad Sci USA.

[20] Genzer Y, Dadon M, Burg C, Chapnik N, Froy O (2015). Ketogenic diet delays the phase of circadian rhythms and does not affect AMP-activated protein kinase (AMPK) in mouse liver. Mol Cell Endocrinol.

[21] Cai Z, Yan LJ, Li K, Quazi SH, Zhao B (2012). Roles of AMP-activated protein kinase in Alzheimer's disease. Neuromolecular Med.

[22] Perluigi M, Di Domenico F, Butterfield DA (2015). mTOR signaling in aging and neurodegeneration: at the crossroad between metabolism dysfunction and impairment of autophagy. Neurobiol Dis.

[23] Siman R, Cocca R, Dong Y (2015). The mTOR inhibitor rapamycin mitigates perforant pathway neurodegeneration and synapse loss in a mouse model of early-stage alzheimer-type tauopathy. PLoS One.

[24] Hsuchou H, Pan W, Kastin AJ (2007). The fasting polypeptide FGF21 can enter brain from blood. Peptides.

[25] Tan BK, Hallschmid M, Adya R, Kern W, Lehnert H, Randeva HS (2011). Fibroblast growth factor 21 (FGF21) in human cerebrospinal fluid: relationship with plasma FGF21 and body adiposity. Diabetes.

[26] Sa-Nguanmoo P, Chattipakorn N, Chattipakorn SC (2016). Potential roles of fibroblast growth factor 21 in the brain. Metab Brain Dis.

[27] Mäkelä J, Tselykh TV, Maiorana F, Eriksson O, Do HT, Mudò G, Korhonen LT, Belluardo N, Lindholm D (2014). Fibroblast growth factor-21 enhances mitochondrial functions and increases the activity of PGC-1α in human dopaminergic neurons via Sirtuin-1. Springerplus.

[28] Tang Z, Bereczki E, Zhang H, Wang S, Li C, Ji X, Branca RM, Lehtiö J, Guan Z, Filipcik P, Xu S, Winblad B, Pei JJ (2013). Mammalian target of rapamycin (mTor) mediates tau protein dyshomeostasis: implication for Alzheimer disease. J Biol Chem.

[29] Roses AD (1994). Apolipoprotein E affects the rate of Alzheimer disease expression: beta-amyloid burden is a secondary consequence dependent on APOE genotype and duration of disease. J Neuropathol Exp Neurol.

[30] Genis I, Gordon I, Sehayek E, Michaelson DM (1995). Phosphorylation of tau in apolipoprotein E-deficient mice. Neurosci Lett.

[31] Gordon I, Grauer E, Genis I, Sehayek E, Michaelson DM (1995). Memory deficits and cholinergic impairments in apolipoprotein E-deficient mice. Neurosci Lett.

[32] Cai H, Cong WN, Ji S, Rothman S, Maudsley S, Martin B (2012). Metabolic dysfunction in Alzheimer's disease and related neurodegenerative disorders. Curr Alzheimer Res.

[33] Masliah E, Mallory M, Ge N, Alford M, Veinbergs I, Roses AD (1995). Neurodegeneration in the central nervous system of apoE-deficient mice. Exp Neurol.

[34] Reitman ML (2007). FGF21: a missing link in the biology of fasting. Cell Metab.

[35] Berger J, Moller DE (2002). The mechanisms of action of PPARs. Annu Rev Med.

[36] Noh HS, Kim YS, Choi WS (2008). Neuroprotective effects of the ketogenic diet. Epilepsia.

[37] Krikorian R, Shidler MD, Dangelo K, Couch SC, Benoit SC, Clegg DJ (2012). Dietary ketosis enhances memory in mild cognitive impairment. Neurobiol Aging.

[38] Mandrekar-Colucci S, Landreth GE (2011). Nuclear receptors as therapeutic targets for Alzheimer's disease. Expert Opin Ther Targets.

[39] Leng Y, Wang Z, Tsai LK, Leeds P, Fessler EB, Wang J, Chuang DM (2015). FGF-21, a novel metabolic regulator, has a robust neuroprotective role and is markedly elevated in neurons by mood stabilizers. Mol Psychiatry.

[40] Bookout AL, de Groot MH, Owen BM, Lee S, Gautron L, Lawrence HL, Ding X, Elmquist JK, Takahashi JS, Mangelsdorf DJ, Kliewer SA (2013). FGF21 regulates metabolism and circadian behavior by acting on the nervous system. Nat Med.

[41] Haigis MC, Sinclair DA (2010). Mammalian sirtuins: biological insights and disease relevance. Annu Rev Pathol.

[42] Kahn BB, Alquier T, Carling D, Hardie DG (2005). AMP-activated protein kinase: ancient energy gauge provides clues to modern understanding of metabolism. Cell Metab.

[43] Vellai T, Takacs-Vellai K, Zhang Y, Kovacs AL, Orosz L, Müller F (2003). Genetics: influence of TOR kinase on lifespan in C. elegans. Nature.

[44] Caccamo A, Magrì A, Medina DX, Wisely EV, López-Aranda MF, Silva AJ, Oddo S (2013). mTOR regulates tau phosphorylation and degradation: implications for Alzheimer's disease and other tauopathies. Aging Cell.

[45] Cai Z, Yan LJ (2013). Rapamycin, Autophagy, and Alzheimer's Disease. J Biochem Pharmacol Res.

[46] Ma L, Dong W, Wang R, Li Y, Xu B, Zhang J, Zhao Z, Wang Y (2015). Effect of caloric restriction on the SIRT1/mTOR signaling pathways in senile mice. Brain Res Bull.

[47] Guo W, Qian L, Zhang J, Zhang W, Morrison A, Hayes P, Wilson S, Chen T, Zhao J (2011). Sirt1 overexpression in neurons promotes neurite outgrowth and cell survival through inhibition of the mTOR signaling. J Neurosci Res.

[48] Stein S, Schäfer N, Breitenstein A, Besler C, Winnik S, Lohmann C, Heinrich K, Brokopp CE, Handschin C, Landmesser U, Tanner FC, Lüscher TF, Matter CM (2010). SIRT1 reduces endothelial activation without affecting vascular function in ApoE−/− mice. Aging (Albany NY).

[49] Palacios OM, Carmona JJ, Michan S, Chen KY, Manabe Y, Ward JL, Goodyear LJ, Tong Q (2009). Diet and exercise signals regulate SIRT3 and activate AMPK and PGC-1alpha in skeletal muscle. Aging (Albany NY).

[50] Mandrekar-Colucci S, Karlo JC, Landreth GE (2012). Mechanisms underlying the rapid peroxisome proliferator-activated receptor-γ-mediated amyloid clearance and reversal of cognitive deficits in a murine model of Alzheimer's disease. J Neurosci.

